# Supplementary material for: Shorter self-reported sleep duration is associated with worse virtual spatial navigation performance in men
Source: Sci Rep. 2024 Feb 19;14:4093. doi: 10.1038/s41598-024-52662-8 (PMC10876962; doi:10.1038/s41598-024-52662-8)
Supplement: Supplementary file 1 — Supplementary Information. [file 41598_2024_52662_MOESM1_ESM.docx]

**Supplementary Materials**

| **Variable** | ***VIF*** |
| --- | --- |
| **Age** | 1.50 |
| **Male gender** | 1.36 |
| **Difficulty waking up** | 3.30 |
| **Sleep duration (hours)** | 2.14 |
| **Sleep inertia** | 5.01 |
| **Sleep quality** | 2.67 |
| **Time spent awake during the night (mins)** | 1.97 |
| **Sleepiness resolution index** | 4.26 |
| **Male gender*Difficulty waking up** | 3.27 |
| **Male gender*Sleep duration (hours)** | 2.08 |
| **Male gender*Sleep inertia** | 4.68 |
| **Male gender*Sleep quality** | 2.64 |
| **Male gender*Time spent awake during the night (mins)** | 1.88 |
| **Male gender*Sleepiness resolution index** | 3.87 |
| **Weekly hours of video gaming on all devices** | 1.30 |
| **Weekly hours of phone use** | 1.12 |
| **Daily hours of sunlight** | 1.11 |
| **Highest level of education achieved** | 1.17 |
| **BMI** | 1.16 |
| **Weekly units of alcohol** | 1.14 |
| **Daily cups of caffeine** | 1.19 |
| **Smoking frequency** | 1.22 |
| **Frequency of daily significant physical activity** | 1.07 |

**Table S1. Variance inflation factor (*VIF*) values for each of the predictor variables included in the main model when specifying sleep duration as a linear term.**

| **Variable** | ***VIF*** |
| --- | --- |
| **Age** | 1.48 |
| **Male gender** | 1.90 |
| **Difficulty waking up** | 3.28 |
| **Sleep duration (hours)** | 1.79 |
| **Sleep inertia** | 5.02 |
| **Sleep quality** | 2.56 |
| **Time spent awake during the night (mins)** | 1.97 |
| **Sleepiness resolution index** | 4.24 |
| **Male gender*Difficulty waking up** | 3.24 |
| **Male gender*Sleep duration (hours)** | 2.42 |
| **Male gender*Sleep inertia** | 4.63 |
| **Male gender*Sleep quality** | 2.61 |
| **Male gender*Time spent awake during the night (mins)** | 1.87 |
| **Male gender*Sleepiness resolution index** | 3.84 |
| **Weekly hours of video gaming on all devices** | 1.30 |
| **Weekly hours of phone use** | 1.12 |
| **Daily hours of sunlight** | 1.11 |
| **Highest level of education achieved** | 1.17 |
| **BMI** | 1.16 |
| **Weekly units of alcohol** | 1.14 |
| **Daily cups of caffeine** | 1.19 |
| **Smoking frequency** | 1.22 |
| **Frequency of daily significant physical activity** | 1.07 |

**Table S2. Variance inflation factor (*VIF*) values for each of the predictor variables included in the main model when specifying sleep duration as a quadratic term.**

**
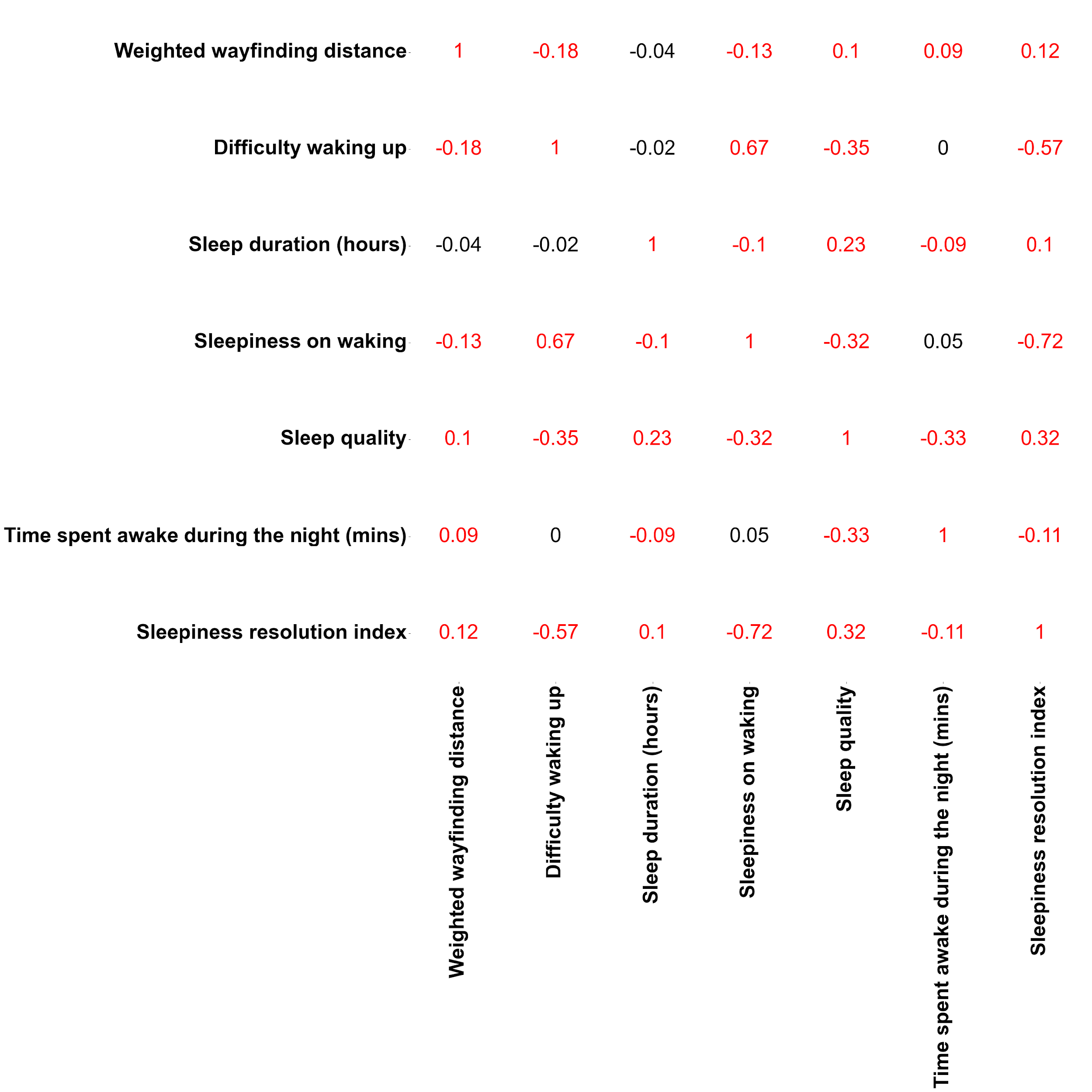
**

**Figure S1. Associations between each of the sleep variables included in the final model and wayfinding distance.** Spearman’s correlation coefficients for the associations between each of the sleep variables (sleep quality, sleep inertia, sleepiness resolution index, sleep duration (hours), time spent awake during the night and difficulty waking up) included in the model and wayfinding distance (across game levels across participants). Values highlighted in red represent significant associations (*p* < 0.05).

**
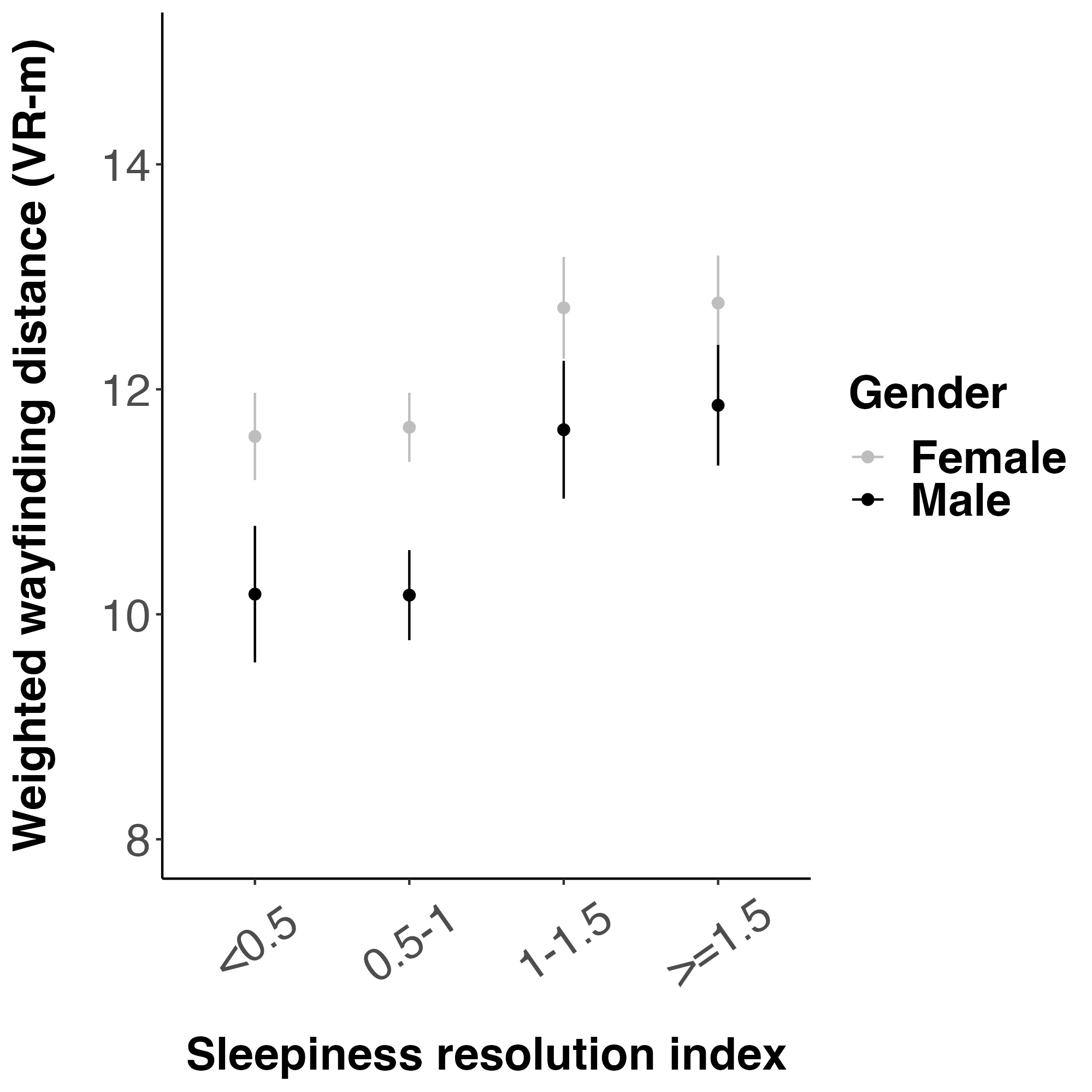

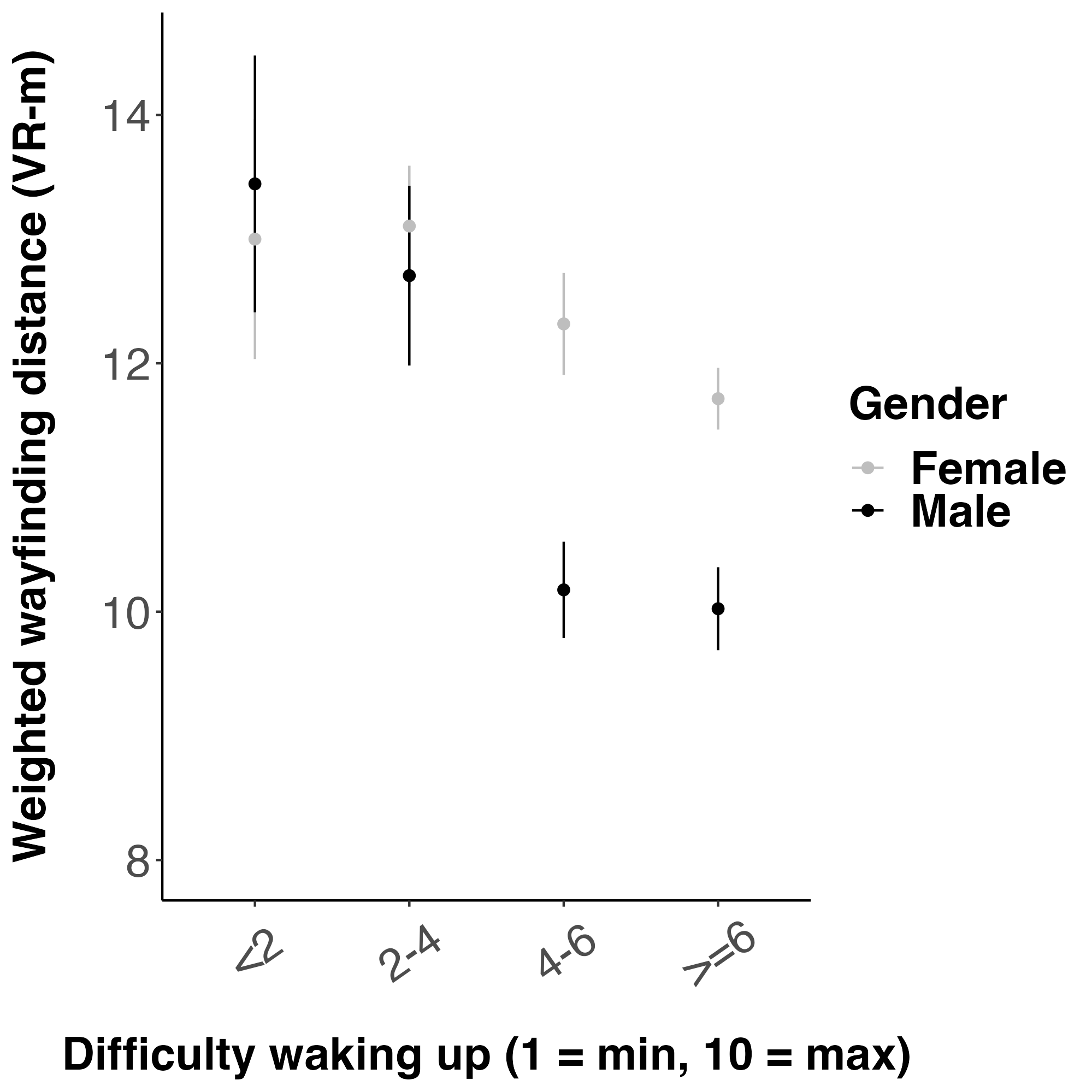
**

**A) B)**

**
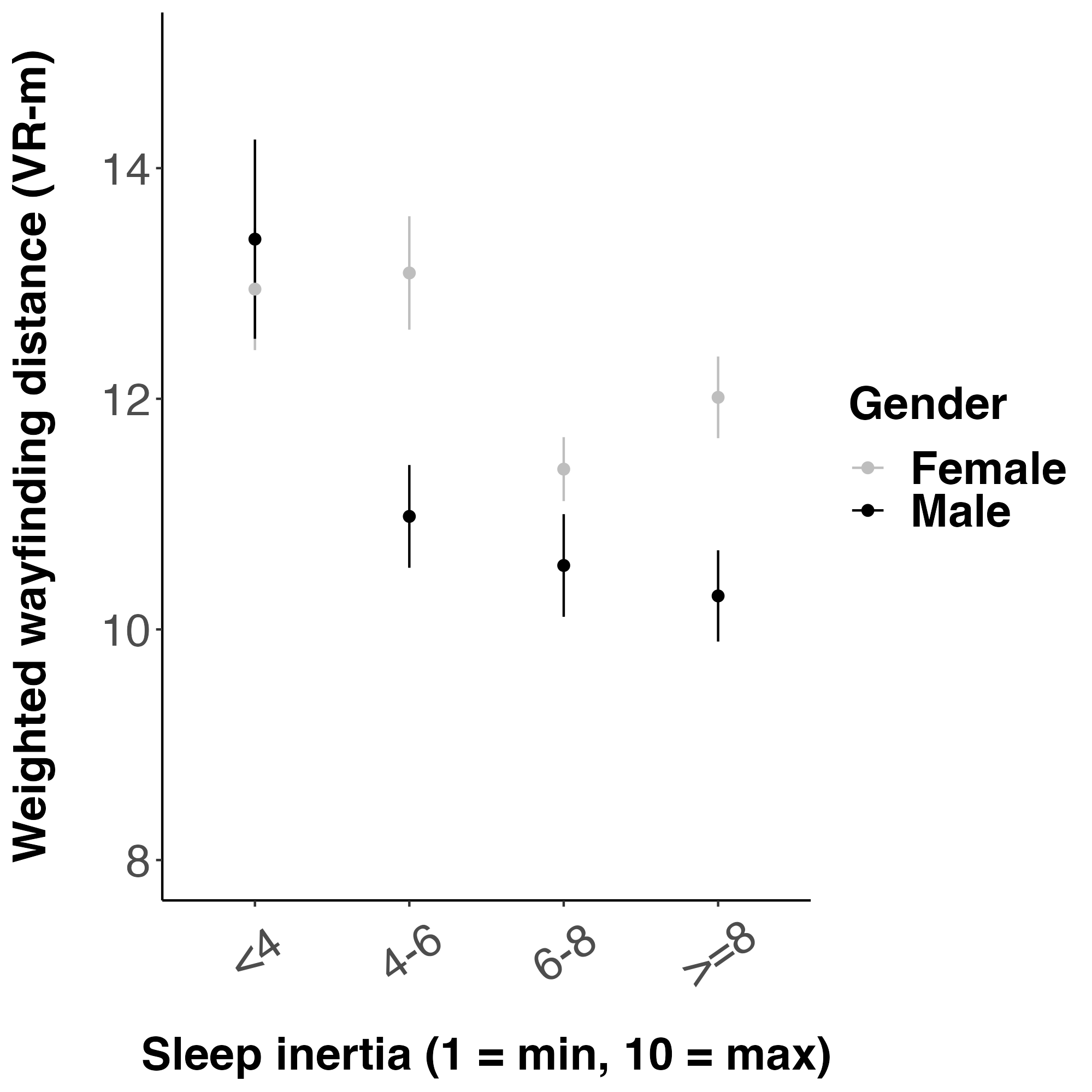

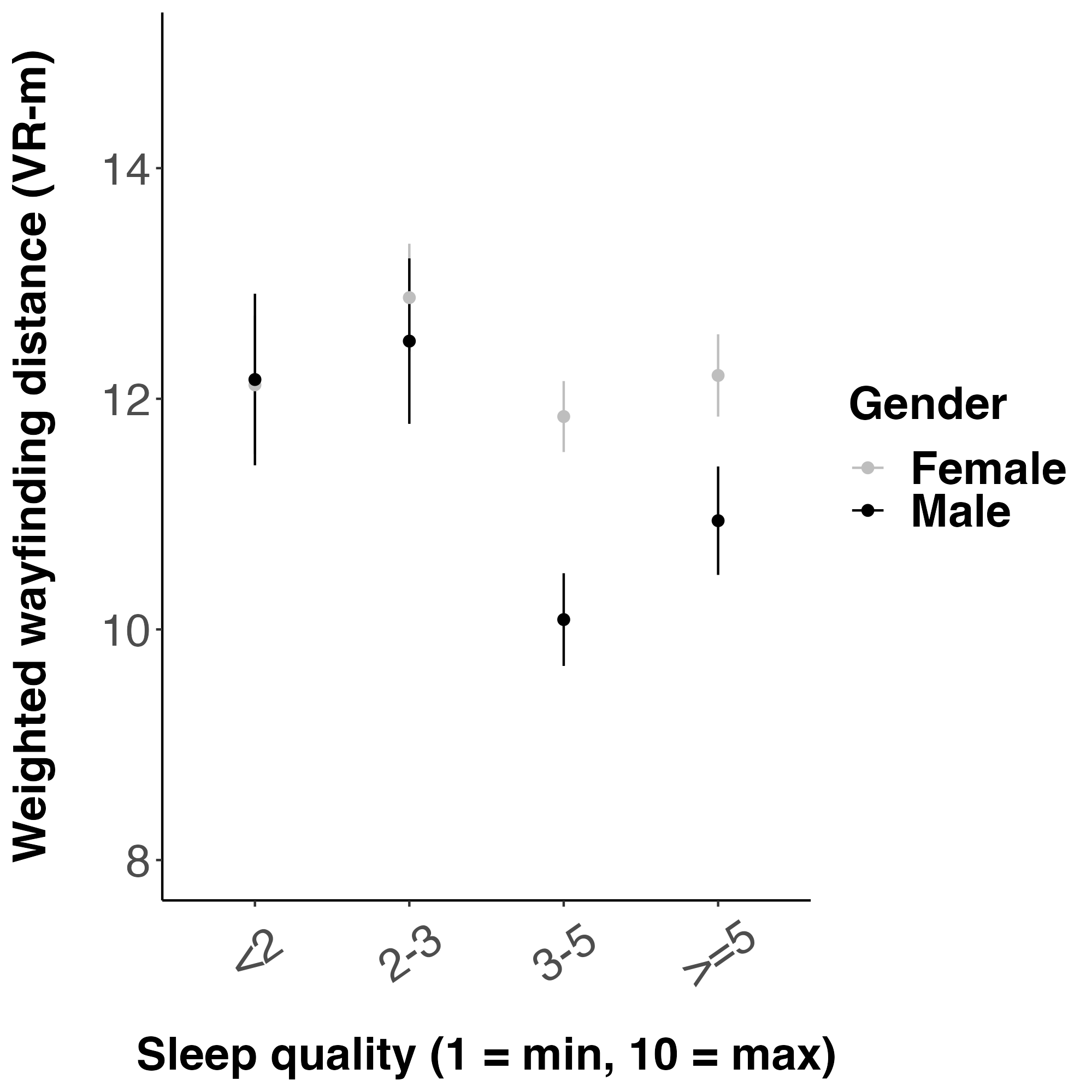
**

**C) D)**

**
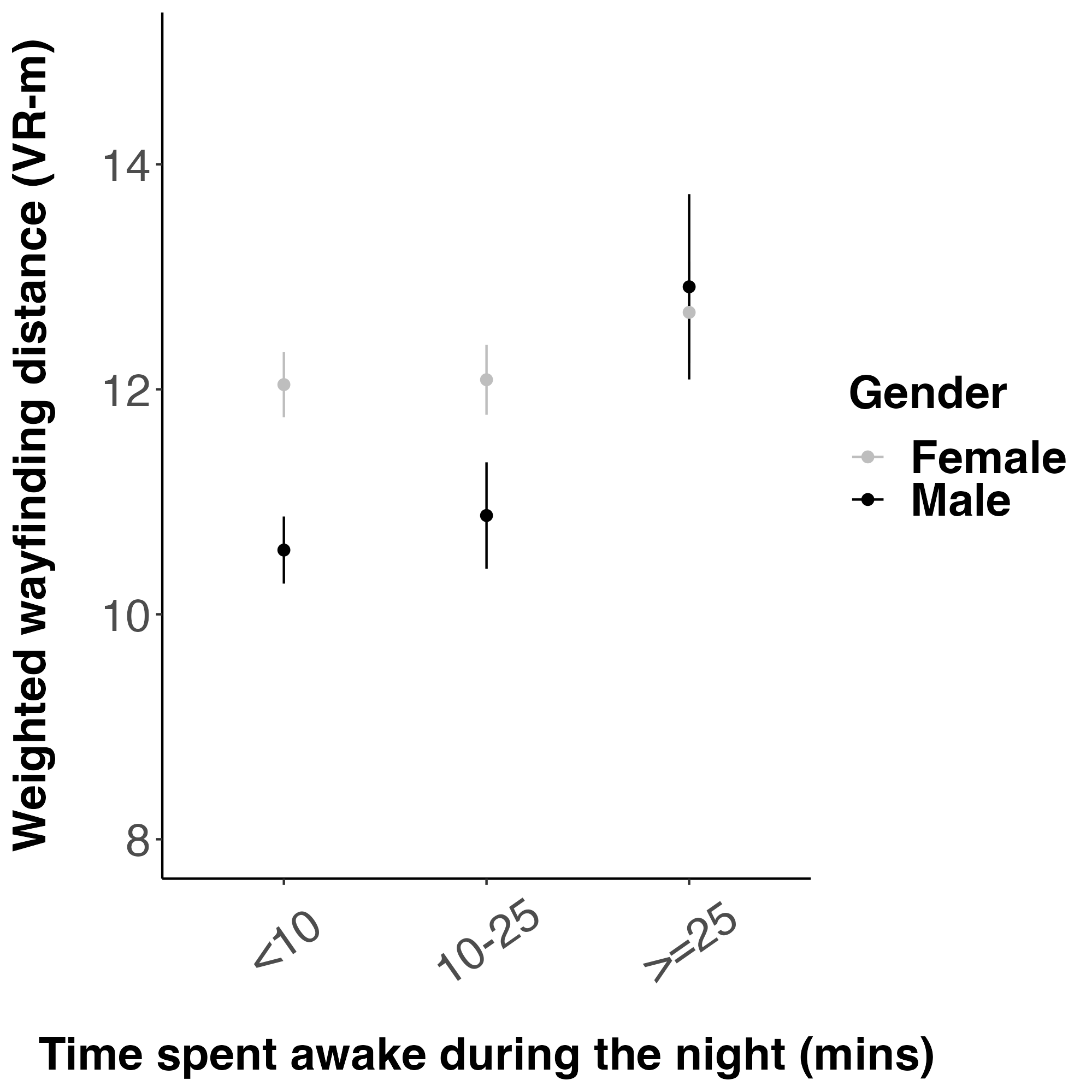
**

**E)**

**Figure S2 Associations between the self-reported sleep characteristics included in the multiple regression model and weighted wayfinding distance.** (A) A value of 1 indicates that the level of sleepiness when going to bed = the level of sleepiness when waking up, a value exceeding 1 indicates that the level of sleepiness on going to bed > the level of sleepiness when waking up and a value of less than 1 indicates that the level of sleepiness on going to bed < level of sleepiness when waking up. (B) 1 = minimum difficulty waking up, 10 = maximum difficulty waking up. (C) 1 = minimum sleep inertia, 10 = maximum sleep inertia. (D) 1 = minimum sleep quality, 10 = maximum sleep quality. (A-D) Data points represent the mean wayfinding distance across game levels across participants. Bars represent one standard error above and below the mean wayfinding distance across game levels across participants.

| **Sleep duration** | ***t*** | ***p*** |
| --- | --- | --- |
| **5** | 0.22 | 0.824 |
| **6** | -1.18 | 0.240 |
| **7** | -1.69 | 0.092 |
| **8** | -3.50 | 0.001 |
| **9+** | -1.58 | 0.121 |

**Table S3. Post-hoc tests comparing weighted wayfinding distance in men and women at each sleep duration.** P-values for the significant differences are highlighted in bold. Negative t-scores indicate greater performance in men than women.

| **Variable** | ***VIF*** |
| --- | --- |
| **Age** | 1.59 |
| **Difficulty waking up** | 2.00 |
| **Sleep duration (hours)** | 1.15 |
| **Sleep inertia** | 2.67 |
| **Sleep quality** | 1.42 |
| **Time spent awake during the night (mins)** | 1.16 |
| **Sleepiness resolution index** | 1.95 |
| **Weekly hours of video gaming on all devices** | 1.10 |
| **Weekly hours of phone use** | 1.15 |
| **Daily hours of sunlight** | 1.20 |
| **Highest level of education achieved** | 1.17 |
| **BMI** | 1.16 |
| **Weekly units of alcohol** | 1.20 |
| **Daily cups of caffeine** | 1.19 |
| **Smoking frequency** | 1.23 |
| **Frequency of daily significant physical activity** | 1.06 |

**Table S4. Variance inflation factors for each of the predictor variables included in the model predicting weighted wayfinding distance using sleep-related variables in men only.**

| **Variable** | ***β*** | ***95% CI*** | ***t*** | ***p*** | **sig** | ***f2*** |
| --- | --- | --- | --- | --- | --- | --- |
| **(Intercept)** | -0.06 | [-0.23, 0.10] | -0.77 | 0.441 |  |  |
| **Age** | 0.15 | [0.05, 0.24] | 3.09 | 0.002 | ** | 0.08 |
| **Difficulty waking up** | -0.10 | [-0.20, 0.01] | -1.78 | 0.076 | . | 0.06 |
| **Sleep duration (hours)** | -0.10 | [-0.18, -0.02] | -2.42 | 0.016 | * | 0.02 |
| **Sleep inertia** | -0.06 | [-0.18, 0.06] | -0.93 | 0.355 |  | 0.01 |
| **Sleep quality** | 0.09 | [<0.01, 0.18] | 1.90 | 0.059 | . | 0.02 |
| **Time spent awake during the night (mins)** | 0.06 | [-0.02, 0.14] | 1.38 | 0.169 |  | 0.01 |
| **Sleepiness resolution index** | -0.04 | [-0.14, 0.07] | -0.70 | 0.483 |  | <0.01 |
| **Weekly hours of video gaming on all devices** | -0.11 | [-0.18, -0.03] | -2.64 | 0.009 | ** | 0.03 |
| **Weekly hours of phone use** | 0.12 | [0.04, 0.20] | 2.92 | 0.004 | ** | 0.04 |
| **Daily hours of sunlight** | 0.03 | [-0.06, 0.11] | 0.63 | 0.528 |  | <0.01 |
| **Highest level of education achieved** | 0.14 | [-0.02, 0.31] | 1.68 | 0.094 | . | 0.01 |
| **BMI** | -0.14 | [-0.22, -0.06] | -3.34 | 0.001 | *** | 0.04 |
| **Weekly units of alcohol** | 0.05 | [-0.03, 0.13] | 1.19 | 0.235 |  | <0.01 |
| **Daily cups of caffeine** | 0.02 | [-0.07, 0.10] | 0.40 | 0.692 |  | <0.01 |
| **Smoking frequency** | -0.16 | [-0.35, 0.02] | -1.73 | 0.085 | . | 0.01 |
| **Frequency of daily significant physical activity** | -0.19 | [-0.34, -0.03] | -2.40 | 0.017 | * | 0.02 |

**Table S5. Model output for predicting weighted wayfinding distance using sleep-related variables and associated covariates in men only.** P-values for the significant associations are highlighted in bold.

| **Variable** | ***VIF*** |
| --- | --- |
| **Age** | 1.38 |
| **Difficulty waking up** | 1.93 |
| **Sleep duration (hours)** | 1.17 |
| **Sleep inertia** | 2.90 |
| **Sleep quality** | 1.42 |
| **Time spent awake during the night (mins)** | 1.22 |
| **Sleepiness resolution index** | 2.33 |
| **Weekly hours of video gaming on all devices** | 1.12 |
| **Weekly hours of phone use** | 1.08 |
| **Daily hours of sunlight** | 1.07 |
| **Highest level of education achieved** | 1.16 |
| **BMI** | 1.17 |
| **Weekly units of alcohol** | 1.12 |
| **Daily cups of caffeine** | 1.19 |
| **Smoking frequency** | 1.18 |
| **Frequency of daily significant physical activity** | 1.12 |

**Table S6. Variance inflation factors for each of the predictor variables included in the model predicting weighted wayfinding distance using sleep-related variables in women only.**

| **Variable** | ***β*** | ***95% CI*** | ***t*** | ***p*** | **sig** | ***f2*** |
| --- | --- | --- | --- | --- | --- | --- |
| **(Intercept)** | 0.08 | [-0.07, 0.22] | 1.04 | 0.299 |  |  |
| **Age** | 0.11 | [0.04, 0.19] | 3.10 | 0.002 | ** | 0.03 |
| **Difficulty waking up** | -0.06 | [-0.15, 0.02] | -1.44 | 0.152 |  | 0.02 |
| **Sleep duration (hours)** | 0.01 | [-0.06, 0.07] | 0.17 | 0.862 |  | <0.01 |
| **Sleep inertia** | -0.06 | [-0.16, 0.05] | -1.05 | 0.294 |  | <0.01 |
| **Sleep quality** | 0.05 | [-0.02, 0.12] | 1.30 | 0.194 |  | <0.01 |
| **Time spent awake during the night (mins)** | 0.05 | [-0.02, 0.12] | 1.46 | 0.144 |  | 0.01 |
| **Sleepiness resolution index** | -0.03 | [-0.12, 0.07] | -0.54 | 0.589 |  | <0.01 |
| **Weekly hours of video gaming on all devices** | -0.09 | [-0.15, -0.02] | -2.60 | 0.010 | ** | 0.01 |
| **Weekly hours of phone use** | 0.07 | [0.01, 0.13] | 2.15 | 0.032 | * | 0.01 |
| **Daily hours of sunlight** | -0.01 | [-0.08, 0.05] | -0.41 | 0.683 |  | <0.01 |
| **Highest level of education achieved** | -0.07 | [-0.22, 0.08] | -0.92 | 0.358 |  | <0.01 |
| **BMI** | <0.01 | [-0.07, 0.06] | -0.13 | 0.900 |  | <0.01 |
| **Weekly units of alcohol** | <0.01 | [-0.07, 0.06] | -0.05 | 0.961 |  | <0.01 |
| **Daily cups of caffeine** | 0.01 | [-0.06, 0.08] | 0.25 | 0.804 |  | <0.01 |
| **Smoking frequency** | 0.02 | [-0.17, 0.21] | 0.23 | 0.821 |  | <0.01 |
| **Frequency of daily significant physical activity** | 0.06 | [-0.07, 0.19] | 0.85 | 0.393 |  | <0.01 |

**Table S7. Model output for predicting weighted wayfinding distance using sleep-related variables and associated covariates in women only.** P-values for the significant associations are highlighted in bold.

| **Variable** | ***VIF*** |
| --- | --- |
| **Age** | 1.51 |
| **Male gender** | 1.38 |
| **Difficulty waking up** | 3.21 |
| **Sleep duration (hours)** | 2.16 |
| **Sleep inertia** | 5.00 |
| **Sleep quality** | 2.63 |
| **Time spent awake during the night (mins)** | 1.98 |
| **Sleepiness resolution index** | 4.31 |
| **Male gender*Difficulty waking up** | 3.16 |
| **Male gender*Sleep duration (hours)** | 2.07 |
| **Male gender*Sleep inertia** | 4.60 |
| **Male gender*Sleep quality** | 2.62 |
| **Male gender*Time spent awake during the night (mins)** | 1.93 |
| **Male gender*Sleepiness resolution index** | 3.93 |
| **Weekly hours of video gaming on all devices** | 1.34 |
| **Weekly hours of phone use** | 1.15 |
| **Daily hours of sunlight** | 1.13 |
| **Highest level of education achieved** | 1.17 |
| **BMI** | 1.19 |
| **Weekly units of alcohol** | 1.14 |
| **Daily cups of caffeine** | 1.23 |
| **Smoking frequency** | 1.26 |
| **Frequency of daily significant physical activity** | 1.08 |

**Table S8. Variance inflation factors for each of the predictor variables included in the model predicting weighted wayfinding distance using sleep-related variables in those who reported sleeping >= 6 and <= 9 hours on a typical night.**

| **Variable** | ***β*** | ***95% CI*** | ***t*** | ***p*** | **sig** | ***f2*** |
| --- | --- | --- | --- | --- | --- | --- |
| **(Intercept)** | 0.04 | [-0.09, 0.16] | 0.57 | 0.572 |  |  |
| **Age** | 0.12 | [0.06, 0.18] | 3.81 | <0.001 | *** | 0.03 |
| **Male gender** | -0.12 | [-0.24, -0.01] | -2.12 | 0.034 | * | 0.04 |
| **Difficulty waking up** | -0.07 | [-0.15, 0.02] | -1.51 | 0.132 |  | 0.02 |
| **Sleep duration (hours)** | 0.01 | [-0.06, 0.08] | 0.34 | 0.737 |  | <0.01 |
| **Sleep inertia** | -0.01 | [-0.12, 0.09] | -0.26 | 0.798 |  | <0.01 |
| **Sleep quality** | 0.02 | [-0.06, 0.10] | 0.47 | 0.641 |  | 0.01 |
| **Time spent awake during the night (mins)** | 0.02 | [-0.05, 0.09] | 0.61 | 0.545 |  | 0.01 |
| **Sleepiness resolution index** | <0.01 | [-0.10, 0.10] | 0.07 | 0.941 |  | <0.01 |
| **Male gender*Difficulty waking up** | -0.01 | [-0.15, 0.12] | -0.17 | 0.867 |  | <0.01 |
| **Male gender*Sleep duration (hours)** | -0.05 | [-0.16, 0.05] | -1.01 | 0.314 |  | <0.01 |
| **Male gender*Sleep inertia** | -0.03 | [-0.19, 0.13] | -0.33 | 0.744 |  | <0.01 |
| **Male gender*Sleep quality** | 0.07 | [-0.04, 0.18] | 1.21 | 0.228 |  | <0.01 |
| **Male gender*Time spent awake during the night (mins)** | 0.04 | [-0.06, 0.15] | 0.80 | 0.422 |  | <0.01 |
| **Male gender*Sleepiness resolution index** | -0.02 | [-0.16, 0.12] | -0.30 | 0.767 |  | <0.01 |
| **Weekly hours of video gaming on all devices** | -0.12 | [-0.17, -0.06] | -4.03 | <0.001 | *** | 0.02 |
| **Weekly hours of phone use** | 0.10 | [0.04, 0.15] | 3.58 | <0.001 | *** | 0.02 |
| **Daily hours of sunlight** | <0.01 | [-0.05, 0.05] | -0.06 | 0.949 |  | <0.01 |
| **Highest level of education achieved** | 0.04 | [-0.08, 0.15] | 0.62 | 0.536 |  | <0.01 |
| **BMI** | -0.05 | [-0.10, <0.01] | -1.87 | 0.062 | . | 0.01 |
| **Weekly units of alcohol** | 0.01 | [-0.04, 0.06] | 0.38 | 0.701 |  | <0.01 |
| **Daily cups of caffeine** | 0.02 | [-0.04, 0.07] | 0.64 | 0.521 |  | <0.01 |
| **Smoking frequency** | -0.05 | [-0.19, 0.08] | -0.76 | 0.449 |  | <0.01 |
| **Frequency of daily significant physical activity** | -0.07 | [-0.17, 0.03] | -1.36 | 0.176 |  | <0.01 |

**Table S9. Model output for predicting weighted wayfinding distance using sleep-related variables and associated covariates when using a subsample of participants who reported sleeping >= 6 and <= 9 hours on a typical night.** P-values for the significant associations are highlighted in bold.

**
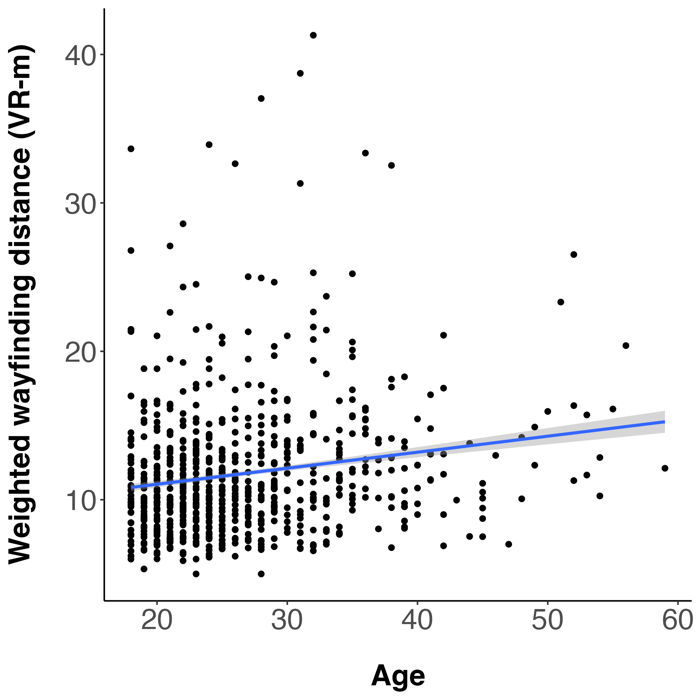
**

**A)**

**
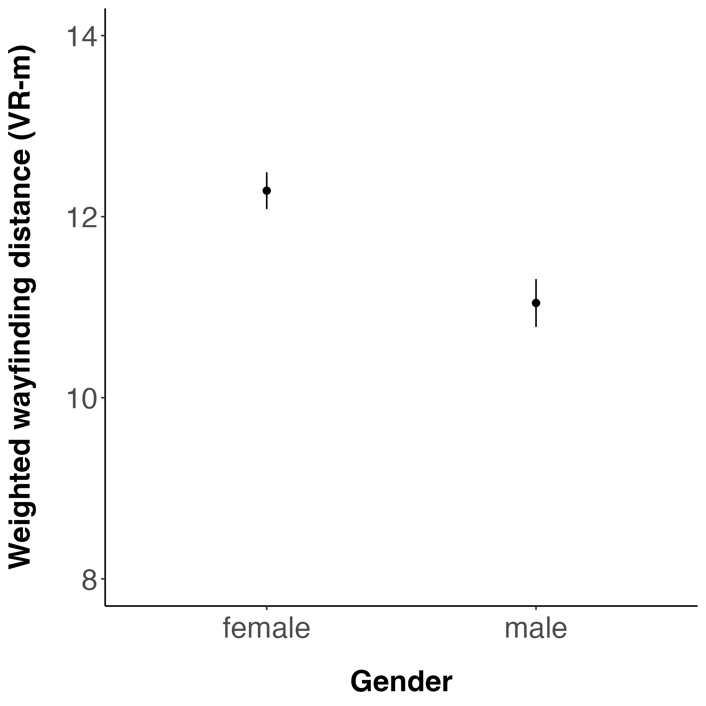
**

**B)**

**
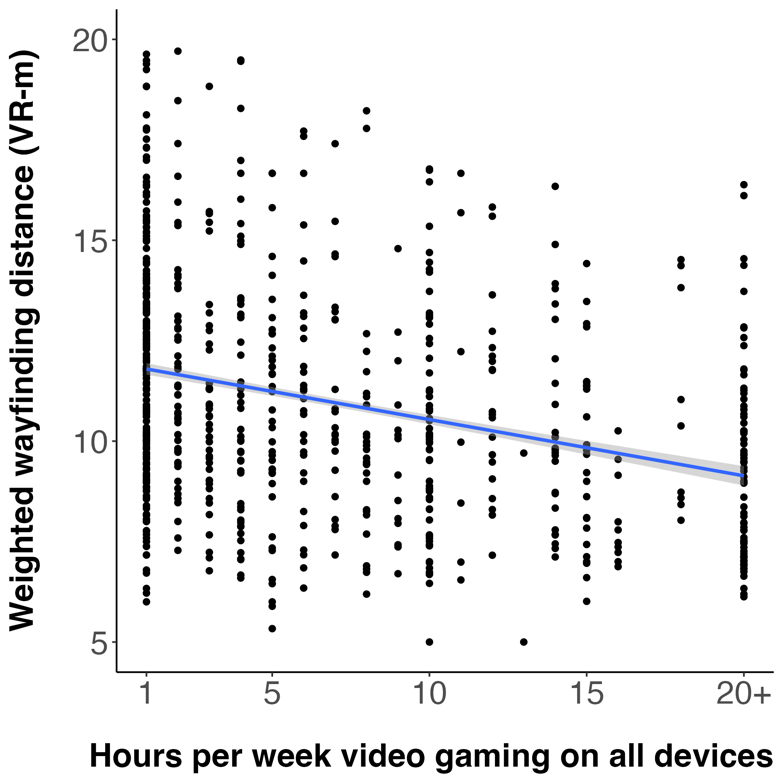
**

**C)**

**
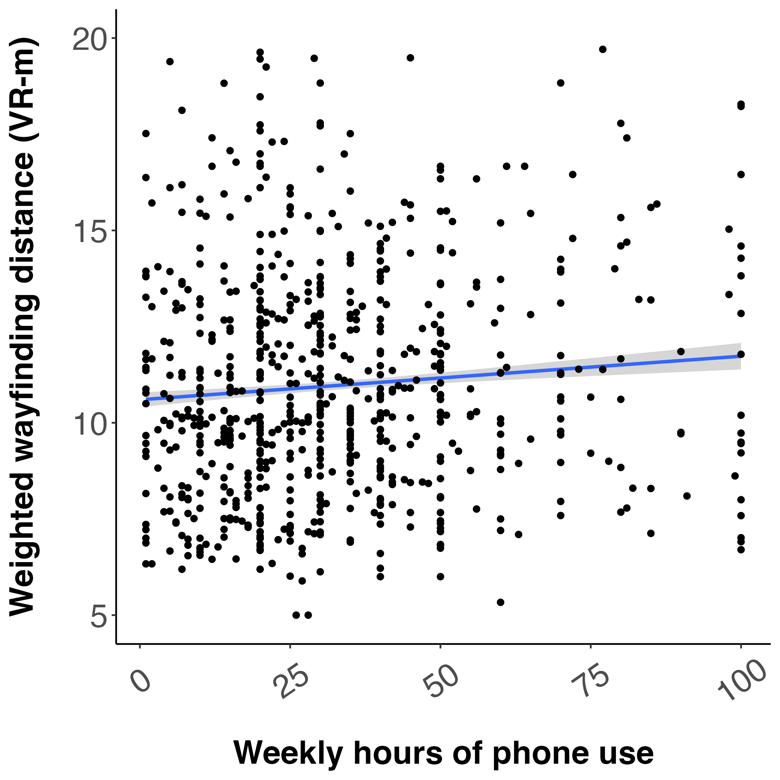
**

**D)**

**
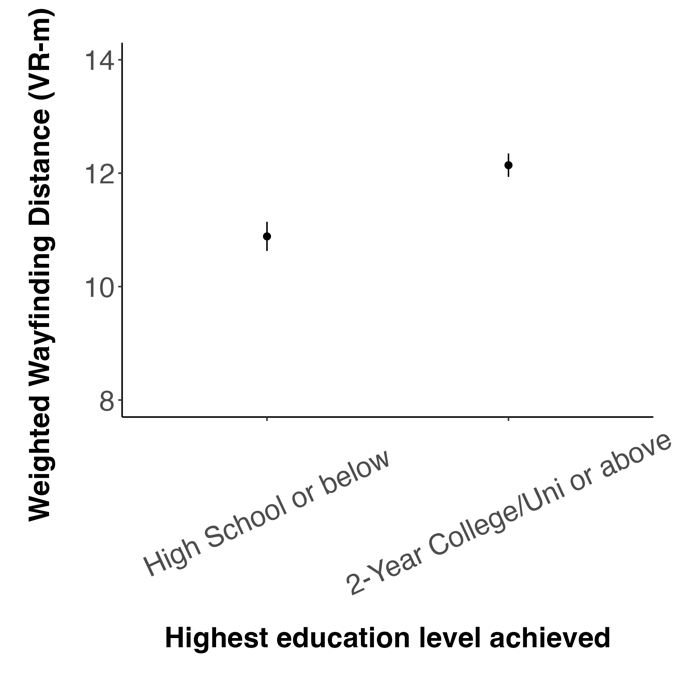
**

**E)**

**
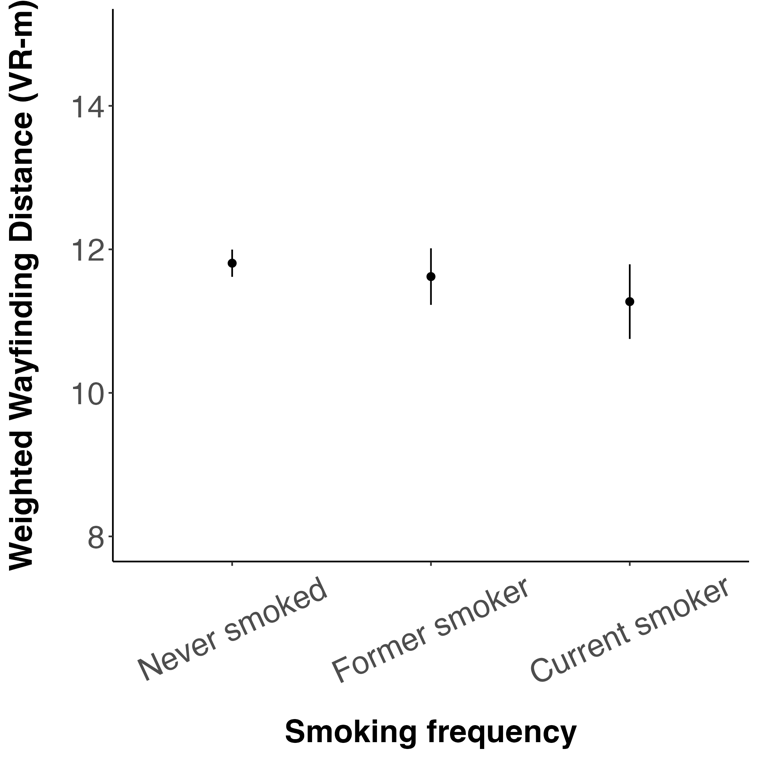
**

**F)**

**
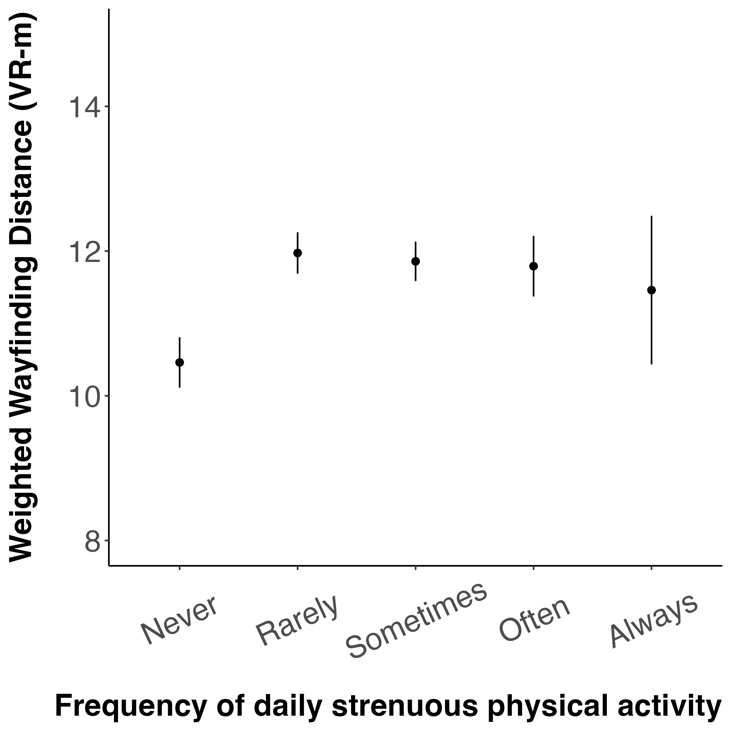
**

**G)**

**
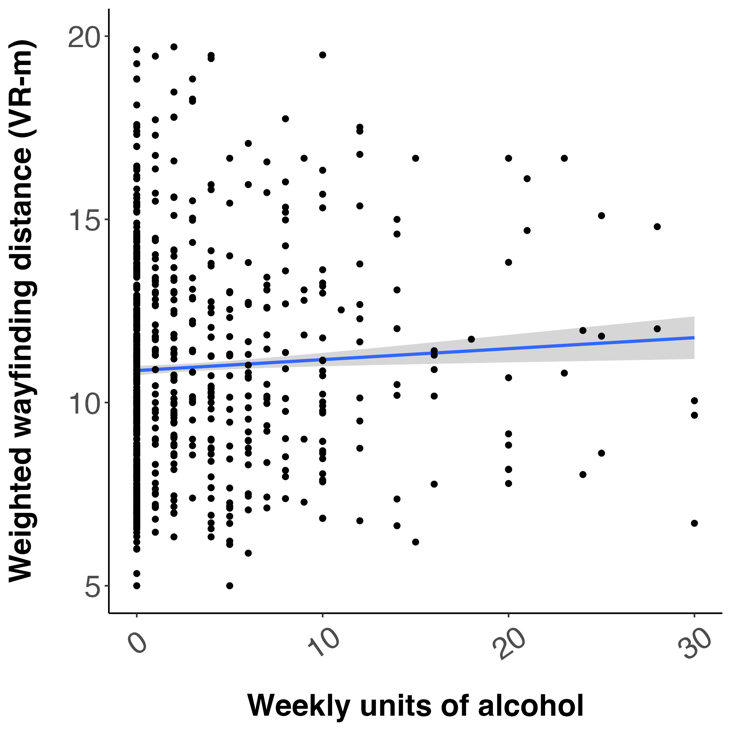
**

**H)**

**
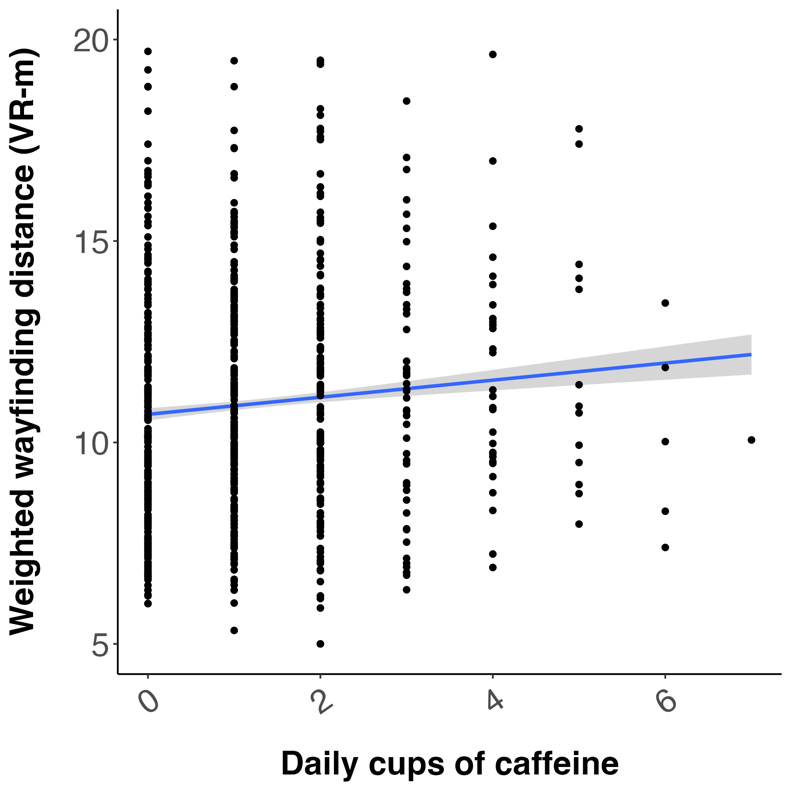
**

**I)**

**
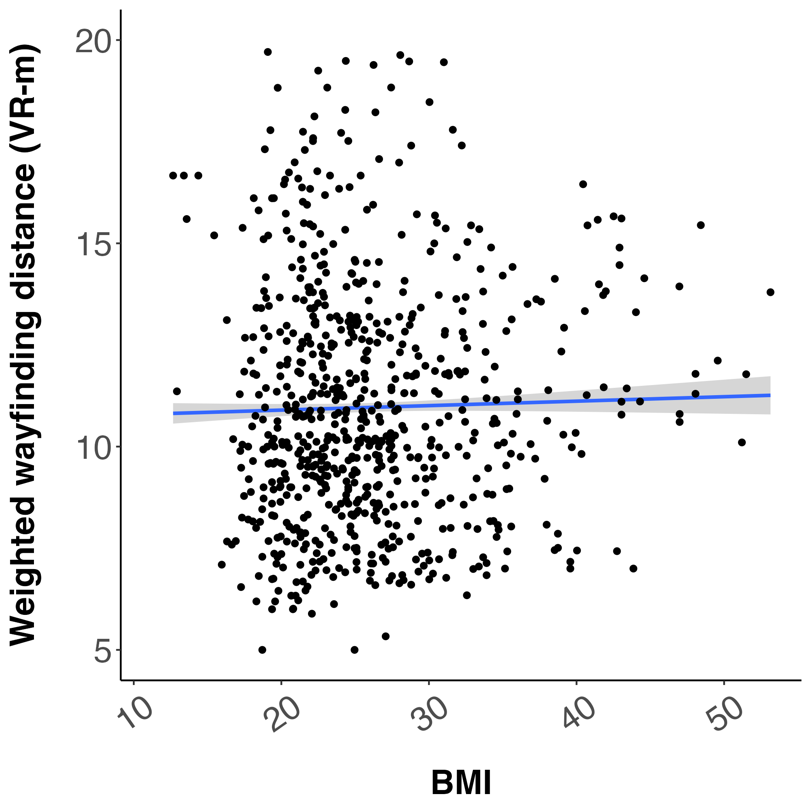
**

**J)**

**
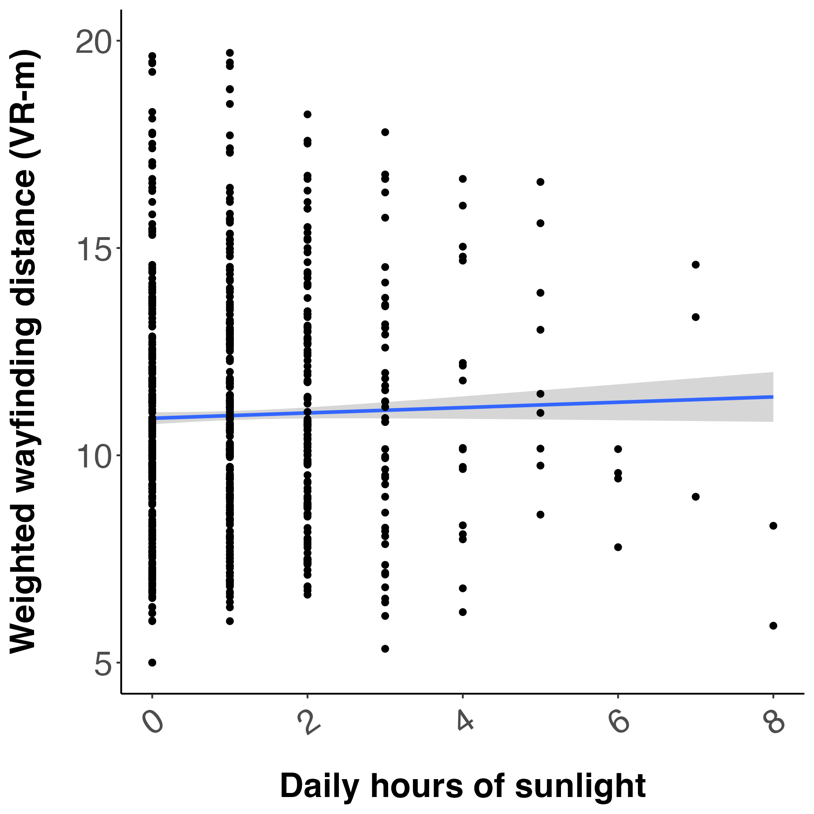
**

**K)**

**Figure S3. (A-K) Associations between each of the demographic variables and weighted wayfinding distance.** VR-m = virtual reality metres

(A, C, D, H-K) Blue line indicates the mean wayfinding distance across game levels across participants. Grey shading surrounding the blue line indicates the standard error of the mean corresponding to this wayfinding distance. Data points indicate the mean wayfinding distance across game levels for an individual participant.

(B, E-G) Data points represent the mean wayfinding distance across game levels across participants. Bars represent the standard error of the mean corresponding to this wayfinding distance.

| **Variable** | ***β*** | ***95% CI*** | ***t*** | ***p*** | **sig** | ***f2*** |
| --- | --- | --- | --- | --- | --- | --- |
| **(Intercept)** | 0.05 | [-0.07, 0.17] | 0.83 | 0.409 |  |  |
| **Age** | 0.12 | [0.06, 0.18] | 4.12 | <0.001 | *** | 0.04 |
| **Male gender** | -0.13 | [-0.24, -0.02] | -2.28 | 0.023 | * | 0.03 |
| **Difficulty waking up** | -0.06 | [-0.15, 0.02] | -1.40 | 0.162 |  | 0.03 |
| **Sleep duration (hours)** | 0.01 | [-0.06, 0.08] | 0.29 | 0.771 |  | <0.01 |
| **Sleep inertia** | -0.04 | [-0.15, 0.07] | -0.75 | 0.452 |  | <0.01 |
| **Sleep quality** | 0.03 | [-0.05, 0.11] | 0.74 | 0.459 |  | 0.01 |
| **Time spent awake during the night (mins)** | 0.05 | [-0.02, 0.11] | 1.32 | 0.187 |  | 0.01 |
| **Sleepiness resolution index** | -0.01 | [-0.11, 0.09] | -0.16 | 0.873 |  | <0.01 |
| **Male gender*Difficulty waking up** | -0.03 | [-0.16, 0.10] | -0.43 | 0.667 |  | <0.01 |
| **Male gender*Sleep duration (hours)** | -0.11 | [-0.22, -0.01] | -2.15 | 0.032 | * | <0.01 |
| **Male gender*Sleep inertia** | -0.03 | [-0.19, 0.13] | -0.43 | 0.671 |  | <0.01 |
| **Male gender*Sleep quality** | 0.07 | [-0.04, 0.18] | 1.18 | 0.238 |  | <0.01 |
| **Male gender*Time spent awake during the night (mins)** | 0.02 | [-0.09, 0.12] | 0.35 | 0.724 |  | <0.01 |
| **Male gender*Sleepiness resolution index** | -0.03 | [-0.17, 0.11] | -0.47 | 0.641 |  | <0.01 |
| **Weekly hours of video gaming on all devices** | -0.11 | [-0.16, -0.05] | -3.84 | <0.001 | *** | 0.02 |
| **Weekly hours of phone use** | 0.10 | [0.05, 0.15] | 3.82 | <0.001 | *** | 0.02 |
| **Weekly hours of phones*Sleep duration (hours)** | -0.02 | [-0.07, 0.03] | -0.85 | 0.396 |  | <0.01 |
| **Daily hours of sunlight** | 0.01 | [-0.04, 0.06] | 0.51 | 0.612 |  | <0.01 |
| **Highest level of education achieved** | 0.04 | [-0.07, 0.15] | 0.73 | 0.466 |  | <0.01 |
| **BMI** | -0.06 | [-0.11, -0.01] | -2.23 | 0.026 | * | 0.01 |
| **Weekly units of alcohol** | 0.02 | [-0.03, 0.08] | 0.93 | 0.351 |  | <0.01 |
| **Daily cups of caffeine** | 0.02 | [-0.03, 0.07] | 0.67 | 0.501 |  | <0.01 |
| **Smoking frequency** | -0.07 | [-0.20, 0.06] | -1.07 | 0.287 |  | <0.01 |
| **Frequency of daily significant physical activity** | -0.06 | [-0.16, 0.04] | -1.19 | 0.236 |  | <0.01 |

**Table S10. Model output for predicting weighted wayfinding distance using sleep-related variables and associated covariates when including an interaction term between hours of sleep duration and weekly hours of phone use.** P-values for the significant associations are highlighted in bold.

| **Variable** | ***r*** | ***p*** | ***95% CI*** |
| --- | --- | --- | --- |
| **Age** | 0.32 | <0.001 | [0.23, 0.41] |
| **Weekly hours of phone use** | <0.01 | 0.961 | [-0.09, 0.09] |
| **Weekly hours of video gaming on all devices** | 0.08 | 0.026 | [-0.01, 0.18] |
| **Sleep duration (hours)** | -0.11 | 0.003 | [-0.20, -0.02] |

**Table S11. Spearman’s correlations showing the associations between BMI and age, hours of sleep, hours of weekly video gaming on all devices and hours of weekly phone use.** P-values highlighted in bold indicate significant associations when applying an alpha threshold of 0.01, bonferroni-corrected with 4 comparisons. *

Questionnaires

**Demographics:**

1. How old are you?

2. What gender are you? (male, female, other)

3. What is the highest level of education you have achieved? (no formal education, some formal education, high school, 2-year college/university, 4-year college/university, masters, PhD)

4. Please indicate your height in feet and inches

5. Please enter your weight in pounds*

*Weight was converted to kg in order to calculate Body Mass Index (BMI)

**Gaming experience:**

1. How often do you play video games per week? (0 = minimum, 20+ hours = maximum)

2. How often you do you use a smartphone or a tablet per week? (0 = minimum, 40+ hours = maximum)

**Sleep-related variables:**

1. What time do you go to sleep? (Please enter the time out of 24:00 hours (00 - 12 AM, 12 - 24 PM)

2. How long does it take you to fall asleep? (Please indicate hours on the left and minutes on the right. For example, if it takes an hour and a half to fall asleep, please enter 01 on the left and 30 on the right).

3. How many times do you wake up on a typical night?

4. How long are you awake for, in total, during the night? (please enter your response in minutes; 1 hr = 60, 2hr = 120, 3hr = 180, 4hr = 240)

5. What time do you wake up? Please enter the time out of 24:00 hours (00 - 12 AM, 12 - 24 PM).

6. Does your alarm clock wake you up? (never, rarely, sometimes, often, always)

7. Do you take naps during the day? (never, rarely, sometimes, often, always)

8. If you take naps during the day, how long is the duration of your nap(s) in total? Please enter your response in minutes. Leave the slider at 0 if you do not take naps. (slider, 0 = min, 240 = max)

9. Do you do any significant physical activity during the day? (never, rarely, sometimes, often, always)

10. How would you rate your sleep quality overall? (1 = very good, 10 = very bad) *

11. How difficult do you find it to wake up/get up? (1 = very easy, 10 = very hard)

12. Please indicate your level of sleepiness upon waking (0 = extremely alert, 10 = extremely sleepy)**

13. Please indicate your level of sleepiness upon bedtime (0 = extremely alert, 10 = extremely sleepy)

14. How many caffeinated beverages do you have a day? (0 = min, 10+ = max)

15. What is the average time you spend outdoors exposed to direct sunlight on typical day? (Please indicate hours on the left and minutes on the right. For example, if you spend an hour and a half in direct sunlight, please enter 01 on the left and 30 on the right)

*This variable was reverse coded (1 = minimum, 10 = maximum)

**This variable is referred to as sleep inertia throughout the manuscript
